# Classifying stages in the gonotrophic cycle of mosquitoes from images using computer vision techniques

**DOI:** 10.1038/s41598-023-47266-7

**Published:** 2023-12-13

**Authors:** Farhat Binte Azam, Ryan M. Carney, Sherzod Kariev, Krishnamoorthy Nallan, Muthukumaravel Subramanian, Gopalakrishnan Sampath, Ashwani Kumar, Sriram Chellappan

**Affiliations:** 1 Dept. of Computer Science and Engineering, University of South Florida, Tampa, FL 33620 USA; 2 Dept. of Integrative Biology, University of South Florida, Tampa, FL 33620 USA; 3 ICMR-Vector Control Research Centre, Field Unit, Madurai, 625002 India; 4 ICMR-Vector Control Research Centre, Puducherry, 605006 India

**Keywords:** Image processing, Machine learning

## Abstract

The ability to distinguish between the abdominal conditions of adult female mosquitoes has important utility for the surveillance and control of mosquito-borne diseases. However, doing so requires entomological training and time-consuming manual effort. Here, we design computer vision techniques to determine stages in the gonotrophic cycle of female mosquitoes from images. Our dataset was collected from 139 adult female mosquitoes across three medically important species—*Aedes aegypti*, *Anopheles stephensi*, and *Culex quinquefasciatus*—and all four gonotrophic stages of the cycle (unfed, fully fed, semi-gravid, and gravid). From these mosquitoes and stages, a total of 1959 images were captured on a plain background via multiple smartphones. Subsequently, we trained four distinct AI model architectures (*ResNet50*, *MobileNetV2*, *EfficientNet-B0*, and *ConvNeXtTiny*), validated them using unseen data, and compared their overall classification accuracies. Additionally, we analyzed t-SNE plots to visualize the formation of decision boundaries in a lower-dimensional space. Notably, *ResNet50* and *EfficientNet-B0* demonstrated outstanding performance with an overall accuracy of 97.44% and 93.59%, respectively. *EfficientNet-B0* demonstrated the best overall performance considering computational efficiency, model size, training speed, and t-SNE decision boundaries. We also assessed the explainability of this *EfficientNet-B0* model, by implementing Grad-CAMs—a technique that highlights pixels in an image that were prioritized for classification. We observed that the highest weight was for those pixels representing the mosquito abdomen, demonstrating that our AI model has indeed learned correctly. Our work has significant practical impact. First, image datasets for gonotrophic stages of mosquitoes are not yet available. Second, our algorithms can be integrated with existing citizen science platforms that enable the public to record and upload biological observations. With such integration, our algorithms will enable the public to contribute to mosquito surveillance and gonotrophic stage identification. Finally, we are aware of work today that uses computer vision techniques for automated mosquito species identification, and our algorithms in this paper can augment these efforts by enabling the automated detection of gonotrophic stages of mosquitoes as well.

## Introduction

Mosquitoes spread many diseases including malaria, dengue, chikungunya, yellow fever, and Zika^1^. In Africa alone, malaria is responsible for the deaths of more than 750,000 people annually, most of them being children^2^. For efforts to model and combat the spread of mosquitoes and mosquito-borne diseases, identifying the gonotrophic stage of the female mosquito is critical for assessing behavior, age, and suitability for different analyses^3,4^. This gonotrophic cycle governs the reproduction of mosquitoes, and consists of four stages. The first is when a female adult is yet to consume a blood meal, the *unfed* stage. When a female mates, it needs to take a blood meal for the eggs to mature. When the mosquito has acquired a full blood meal, its eggs are ready to mature, and the mosquito is in the *fully fed* stage. The next stage is when the eggs have started to mature as the blood is being digested, and this is called the *semi-gravid* or half-gravid stage. When the eggs are fully mature (i.e., when the blood is fully digested), the mosquito is in the *gravid* stage.

**Figure 1:**
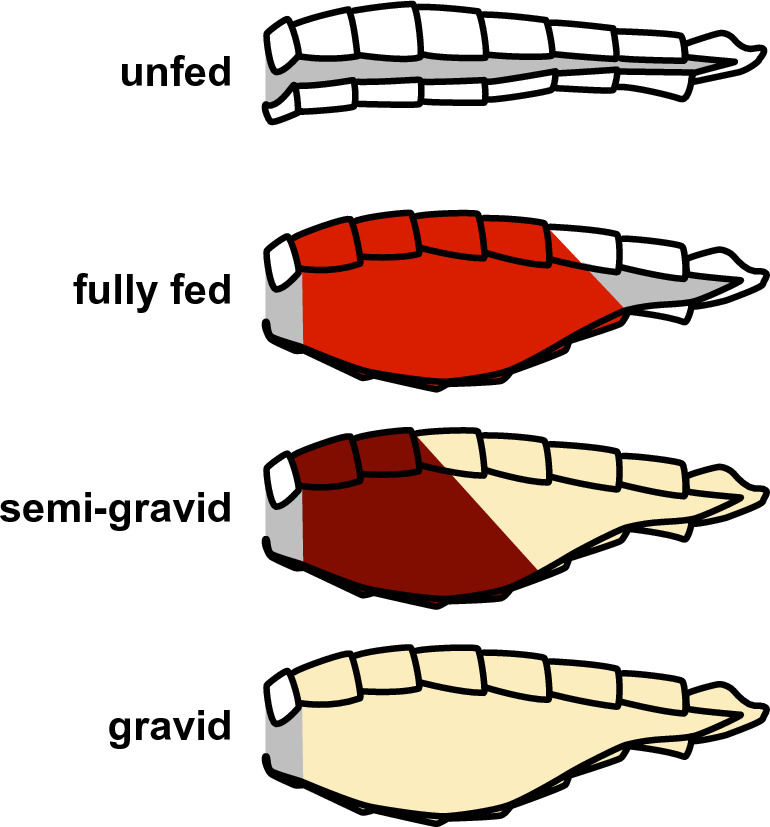
Abdominal conditions of a female mosquito according to the stages of its gonotrophic cycle, redrawn from^4^.

Our goal in this paper is to automate the identification of gonotrophic stages in a female mosquito. Today, this process is manual, time-consuming, and requires trained expertise that is increasingly harder to find. Each mosquito specimen has to be visually analyzed to look for the color and shape of the abdomen to make a determination of the gonotrophic stage (see Fig. 1), and there is a need to automate this process. With automation, mosquito observations from the general public can also be processed, which can provide larger-scale surveillance data for public health agencies.

### Our technical contributions

We raised 97 female mosquitoes in a lab in Southern India and let them go through all four stages in the gonotrophic cycle. The mosquitoes were distributed between three medically important vector species—*Aedes (Ae.) aegypti, Culex (Cx.) quinquefasciatus*, and *Anopheles (An.) stephensi*. Subsequently, as the mosquitoes went through each stage, our team took pictures of them via multiple smartphones on a plain grey or white background to generate a total of 1379 images (details on our dataset are provided in Discussions and Methods sections). In addition, we also raised 42 *Anopheles stephensi* mosquitoes at a lab in the US, from which we took 580 images of these mosquitoes in their unfed and semi-gravid stages via multiple smartphones on a similar background. Our total image dataset was thus 1959 images (see Table 1). Using this dataset, our contributions are the following.

- *Designing multiple neural network architectures for classification:* In this study, we trained, fine-tuned, and tested four different neural network architectures for classifying gonotrophic stages—*ResNet50*^5^, *MobileNetV2*^6^, *EfficientNet-B0*^7^, and *ConvNeXtTiny*^8^. Each architecture provides contextual relevance to our classification problem, while also being diverse from the other in their design. The *ResNet50* is popular, but computationally very expensive. The *MobileNetV2* architecture is lighter and particularly suited for execution on embedded devices like smartphones. The *EfficientNet-B0* architecture is newer and is a nice trade-off between good accuracy and lighter in complexity. Finally, the *ConvNeXtTiny* architecture is a hybrid of CNNs and the more recent Vision Transformers^9^. Our metrics to assess performance were precision, recall, F1-score, and accuracy. Our analysis identified that overall the *EfficientNet-B0* architecture out-performed others. This model was able to yield an overall accuracy of $93.59\%$, with a tolerable model size and speed of execution. Most confusion happened between the gravid and semi-gravid stages across all models.
- *Visualizing the predictive ability of features using t-SNE Analysis*: We leveraged the t-distributed Stochastic Neighbor Embedding (t-SNE) algorithm^10^ to construct a 2D plot to visualize the features extracted by our AI models. We observed that the results obtained from the *EfficientNet-B0* model displayed distinct and separable features for each class, aligning precisely with the different stages of the gonotrophic cycle. This observation further substantiates the effectiveness of the *EfficientNet-B0* model in gonotrophy stage classification.
- *Providing model explainability via Grad-CAMs: * To further the explainability of our trained *EfficientNet-B0* model, we utilized the Gradient-weighted Class Activation Mapping (Grad-CAM)^11^ technique to identify those pixels that the AI model prioritized in making a classification. Our findings demonstrate that our model gives the greatest weight to those pixels that represent the abdomen of the mosquito. This finding is important and indicates that our AI model has learned correctly because the visual markers for identifying stages in the gonotrophic cycle are indeed located in the abdomen of a mosquito (please refer to Fig. 1).
- *Highlighting the practical impact of our work*: To the best of our knowledge, our study is the first to design and validate computer vision methods for automatic identification of the stages in the mosquito gonotrophic cycle. The practical impact of our study is elaborated later in the paper.

**Table 1:** Number of mosquito images and specimens across three species in our dataset.

	*Ae. aegypti*	*An. stephensi*	*Cx. quinquefasciatus*
	Images (specimens)	Images (specimens)	Images (specimens)
Unfed	151 (10)	370 (23)	58 (6)
Fully fed	146 (20)	176 (10)	199 (7)
Semi-gravid	121 (12)	210 (19)	107 (5)
Gravid	118 (12)	164 (9)	139 (6)

## Results

### Classification accuracies

The results here are for 234 images in our testing dataset that are unseen by the four AI models that we trained. They are presented in Table 2. Our metrics to test are Precision, Recall, F1-score, and Accuracy. These metrics are calculated the same way for each class and are defined below:

1 $$\begin{aligned} Precision= & {} \frac{True\;Positive}{True\;Positive + False\;Positive}, \end{aligned}$$

2 $$\begin{aligned} Recall= & {} \frac{True\;Positive}{True\;Positive + False\;Negative}, \end{aligned}$$

3 $$\begin{aligned} F\it{1} \text {-} score= & {} 2 * \frac{Precision * Recall}{Precision + Recall}, \end{aligned}$$

4 $$\begin{aligned} Accuracy= & {} \frac{True\;Positive + True\;Negative}{Positive + Negative}. \end{aligned}$$

As we can see from Table 2, the highest classification accuracy is yielded by the *ResNet50* model, followed by the *EfficientNet-B0* model. The lowest classification accuracy was yielded by the *ConvNeXtTiny* model, which is a new architecture combining the features of CNNs, and drawing inspiration from Vision Transformers^9^. Table 3 presents the confusion matrices for all four architectures. As we can see all models exhibit confusion classifying between semi-gravid and gravid mosquitoes. This is reasonable because the morphological differences between these two classes are very fine – extremely delicate and inexact changes in color across the abdomen of the mosquito – which sometimes confuses even trained entomologists.

To analyze the architectures further, Table 4 presents the complexity of the models trained, since it is also important that models are lightweight and leave minimal footprints in their execution. As we can see the *ResNet50* model is the heaviest with a very large model size and features extracted. The *EfficientNet-B0* model is much lighter in comparison. It is our judgment that for the problem of classifying gonotrophic stages, the *EfficientNet-B0* model is most practical, since it is very accurate, and lightweight also, lending its ability to be executed in embedded devices like smartphones and edge computers, which is the practical need in mosquito surveillance. The average inference time per image for the *ResNet50*, *EfficientNet-B0*, *MobileNetV2*, and *ConvNeXtTiny* models were 0.82, 1,22, 0.67, and 2.58 seconds respectively. These are small and tolerable delays.

**Table 2:** Results for four classification model architectures on test images.

Performance metrics	Unfed	Fully fed	Semi-gravid	Gravid	Avg. performance metrics
ResNet50					
Precision	100	96.08	100	91.84	96.98
Recall	100	94.23	95.45	100	97.42
F1-score	100	95.15	97.67	95.74	97.14
Accuracy	100	94.23	95.45	100	97.44

MobileNetV2					
Precision	95.95	91.84	95.08	80.00	90.71
Recall	100	86.54	87.88	88.89	90.83
F1-score	97.93	89.11	91.34	84.21	90.65
Accuracy	100	86.53	87.88	88.89	91.45

EfficientNet-B0					
Precision	97.26	96.00	95.24	83.33	92.96
Recall	100	92.31	90.91	88.89	93.03
F1-score	98.61	94.12	93.02	90.91	92.94
Accuracy	100	92.31	90.91	88.89	93.59

ConvNeXtTiny					
Precision	94.20	92.31	89.66	69.09	86.31
Recall	91.55	92.31	78.79	84.44	86.77
F1-score	92.86	92.31	83.87	76.00	86.26
Accuracy	91.55	92.31	78.78	84.44	86.75

**Table 3:** Confusion matrices for four classification model architectures on test images.

(a) ResNet50					
		Predicted			
		Unfed	Fully fed	Semi-gravid	gravid
Actual	Unfed	**71**	0	0	0
	Fully fed	0	**49**	0	3
	Semi-gravid	0	2	**63**	1
	Gravid	0	0	0	**45**

(b) MobileNetV2					
		Predicted			
		Unfed	Fully fed	Semi-gravid	Gravid
Actual	Unfed	**71**	0	0	0
	Fully fed	0	**45**	1	6
	Semi-gravid	1	3	**58**	4
	Gravid	2	1	2	**40**

(c) EfficientNet-B0					
		Predicted			
		Unfed	Fully fed	Semi-gravid	Gravid
Actual	Unfed	**71**	0	0	0
	Fully fed	0	**48**	0	4
	Semi-gravid	0	2	**60**	4
	Gravid	2	0	3	**40**

(d) ConvNeXtTiny					
		Predicted			
		Unfed	Fully fed	Semi-gravid	Gravid
Actual	Unfed	**65**	1	0	5
	Fully fed	0	**48**	2	2
	Semi-gravid	3	1	**52**	10
	Gravid	1	2	4	**38**

Correctly predicted instances are in bold.

**Table 4:** Comparison of model architectures from a complexity perspective.

Model name	Number of layers	Number of parameters	Model size	Num. of features extracted from the last convolution layer
ResNet50	188	24,165,764	290.50MB	2048
MobileNetV2	167	1,763,508	21.80MB	1280
EfficientNet-B0	250	4,431,015	51.60MB	1280
ConvNeXtTiny	164	28,070,500	337.80MB	768

### Features visualization using t-distributed stochastic neighbor embedding (t-SNE) algorithm

In order to highlight the discriminatory power across the four classes of gonotrophic stages, we leverage the technique of t-SNE ^10^. Basically, t-SNE is an unsupervised, non-linear technique for dimensionality reduction, and is used for visualizing high-dimensional data (in our case, the activation maps or output features of the final convolutional layer of our AI model). Basically, this method provides an intuition of how high-dimensional data points are related in low-dimensional space. As such, we can use this technique to evaluate the discriminatory power of the AI models.

To implement the t-SNE method, the following steps were executed for all four AI models. For each model, we start with the base, from which two sequential phases were executed. First, t-SNE builds up a probability distribution matrix for data points, which are the activation maps of the final convolutional layer of our AI model. For each pair, if there is a high level of similarity, a large probability value is assigned; otherwise, the probability value is small. Next, t-SNE considers those data points in a lower-dimensional space and generates another probability distribution. Here, the algorithm minimizes the loss or difference between the two probability distributions with respect to the locations on the map. To accomplish that, the algorithm calculates the Kullback-Leibler divergence (KL divergence)^12^ value and minimizes it over several iterations. This helps us understand how our AI model separates different classes in the data by visualizing how the decision boundaries are formed in a lower-dimensional space.

**Figure 2:**
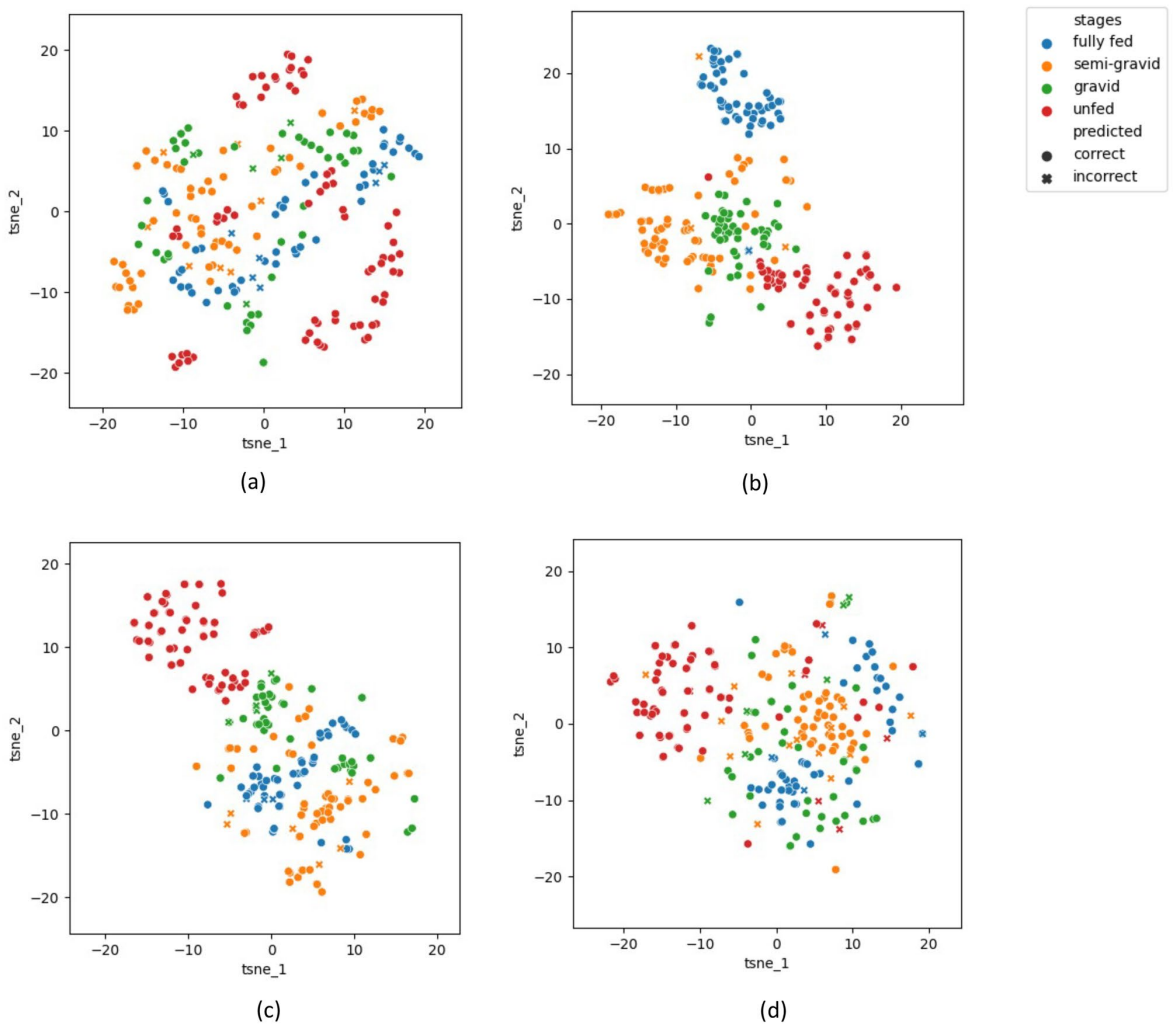
Feature maps of all trained models after implementing the t-SNE algorithm. (**a**) *ResNet50*, (**b**) *MobileNetV2*, (**c**) *EfficientNet-B0*, (**d**) *ConvNeXtTiny*.

For each AI model, we obtained the activation maps from the final convolutional layer for all 234 test images, and each image resulted in a matrix with dimensions of $7 \times 7 \times m$ (where *m* denotes the number of features extracted from the last convolution layer of each model (see Table 4)). To prepare the data for analysis, we flattened each image’s feature matrix into an array of size $49 \times m$. Subsequently, we applied the t-SNE algorithm to the flattened feature data of the 234 images, as described earlier. This process generated 2D coordinates for each image, allowing us to visualize them in a reduced space. To provide additional information, we color-coded the images based on their gonotrophic stages in Fig. 2, and used circles and crosses to denote correct and incorrect classifications.

In Fig. 2, we show the resulting t-SNE plots for all four trained AI models. While *ResNet50* (Fig. 2a) and *ConvNeXtTiny* (Fig. 2d) achieved higher classification accuracies, they did not exhibit a clearly distinguishable t-SNE plot. On the other hand, although *MobileNetV2* (Fig. 2b) displayed a coherent t-SNE plot, this model had the poorest performance metrics for the fully fed class. Notably, the t-SNE plot for *EfficientNet-B0* (Fig. 2c) demonstrated better alignment with its performance metrics for all classes. The markers corresponding to each color (representing a gonotrophic stage) were distinctly located in separate areas on the plot, reaffirming that the AI had effectively learned discernible features, and classified them accurately.

### Enhancing model explainability utilizing Grad-CAMs

In this study, we provide further explainability of our AI model (*EfficientNet-B0* only). In our study, we attempt to do so using the technique of Grad-CAM^11^. Grad-CAM is a technique that leverages the gradients of each target class as they propagate through the final convolutional layer of a neural network. By analyzing these gradients, Grad-CAM generates a coarse localization map that highlights the important regions of an image that contribute to the network’s prediction for a specific class. To accomplish this, Grad-CAM first computes the gradients of the target class with respect to the feature maps produced by the final convolutional layer. These gradients serve as important weights, indicating how crucial each feature map is for predicting the class of interest. Next, the gradients are global-average-pooled to obtain a single weight per feature map. This pooling operation helps to capture the overall importance of each feature map rather than focusing on individual spatial locations. Finally, the weights are combined with the corresponding feature maps using a weighted combination, producing the final localization map. This map provides a visual representation of the regions in the image that are most relevant for the neural network’s decision-making process regarding the target class. In the resulting implementation of this technique, the pixels in an image that were prioritized more during a classification will appear progressively redder, while those pixels prioritized less will appear progressively bluer.

**Figure 3:**
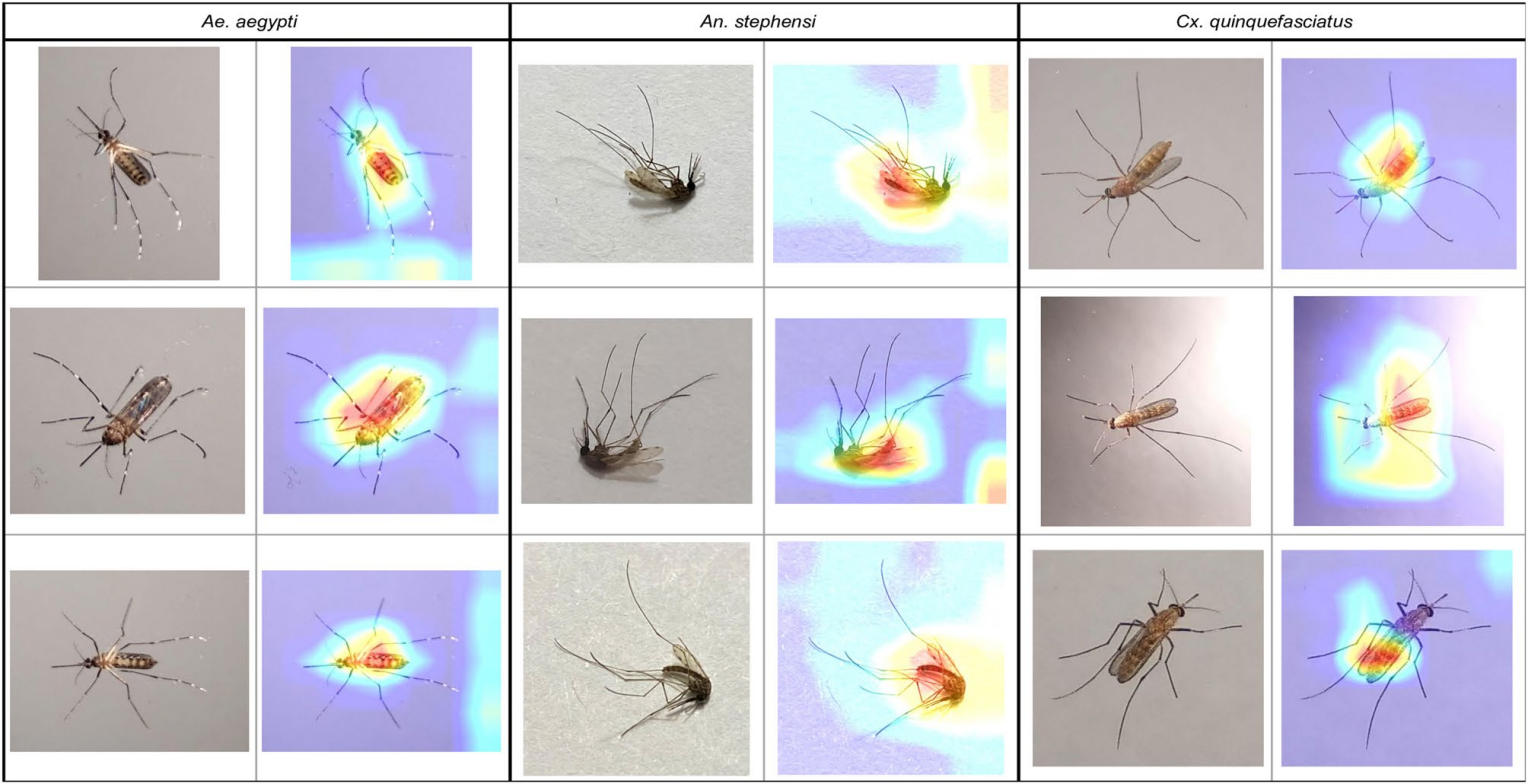
Unfed gonotrophic stage images with Grad-CAM per species for the *EfficientNet-B0* model.

**Figure 4:**
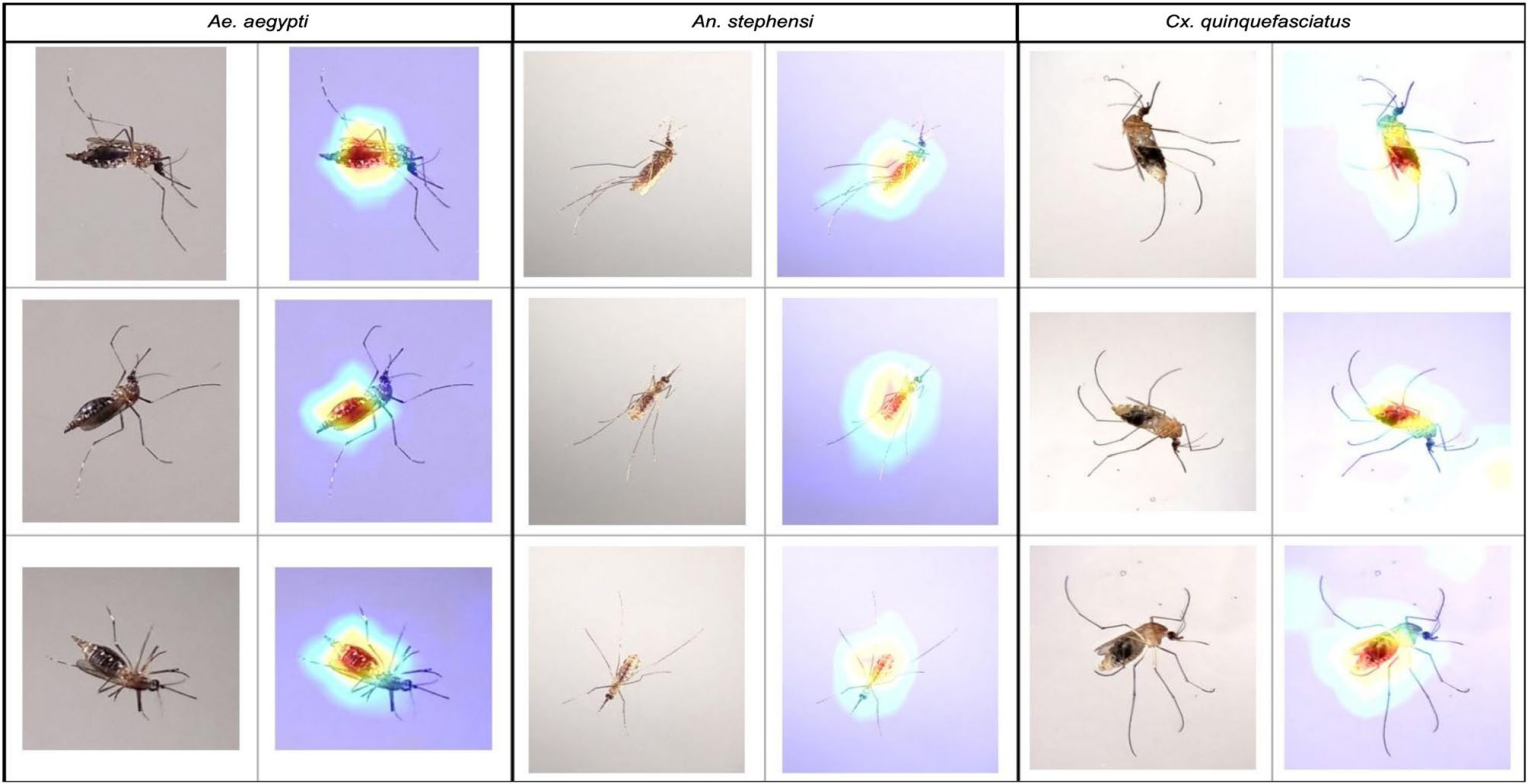
Fully fed gonotrophic stage images with Grad-CAM per species for the *EfficientNet-B0* model.

**Figure 5:**
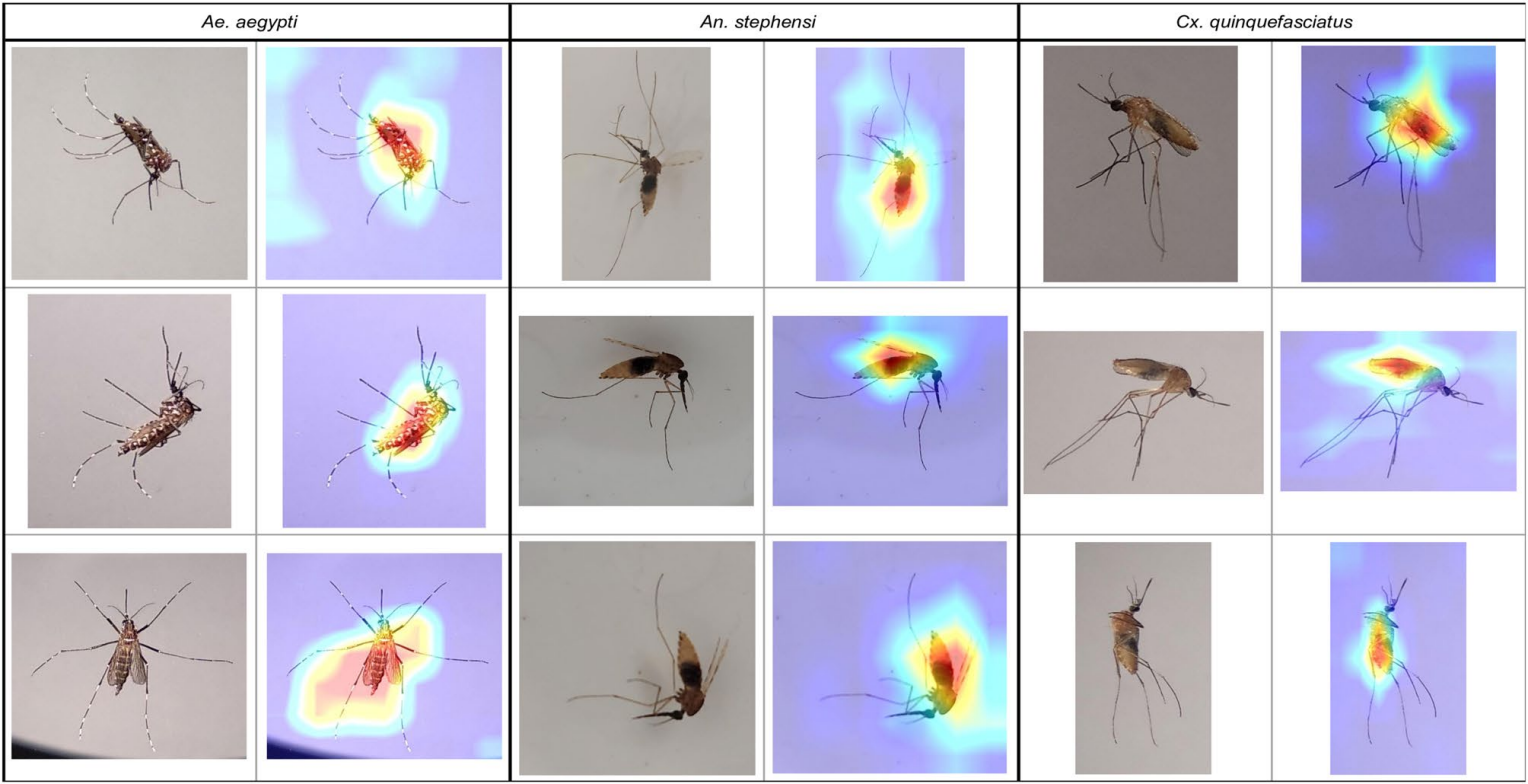
Semi-gravid gonotrophic stage images with Grad-CAM per species for the *EfficientNet-B0* model.

**Figure 6:**
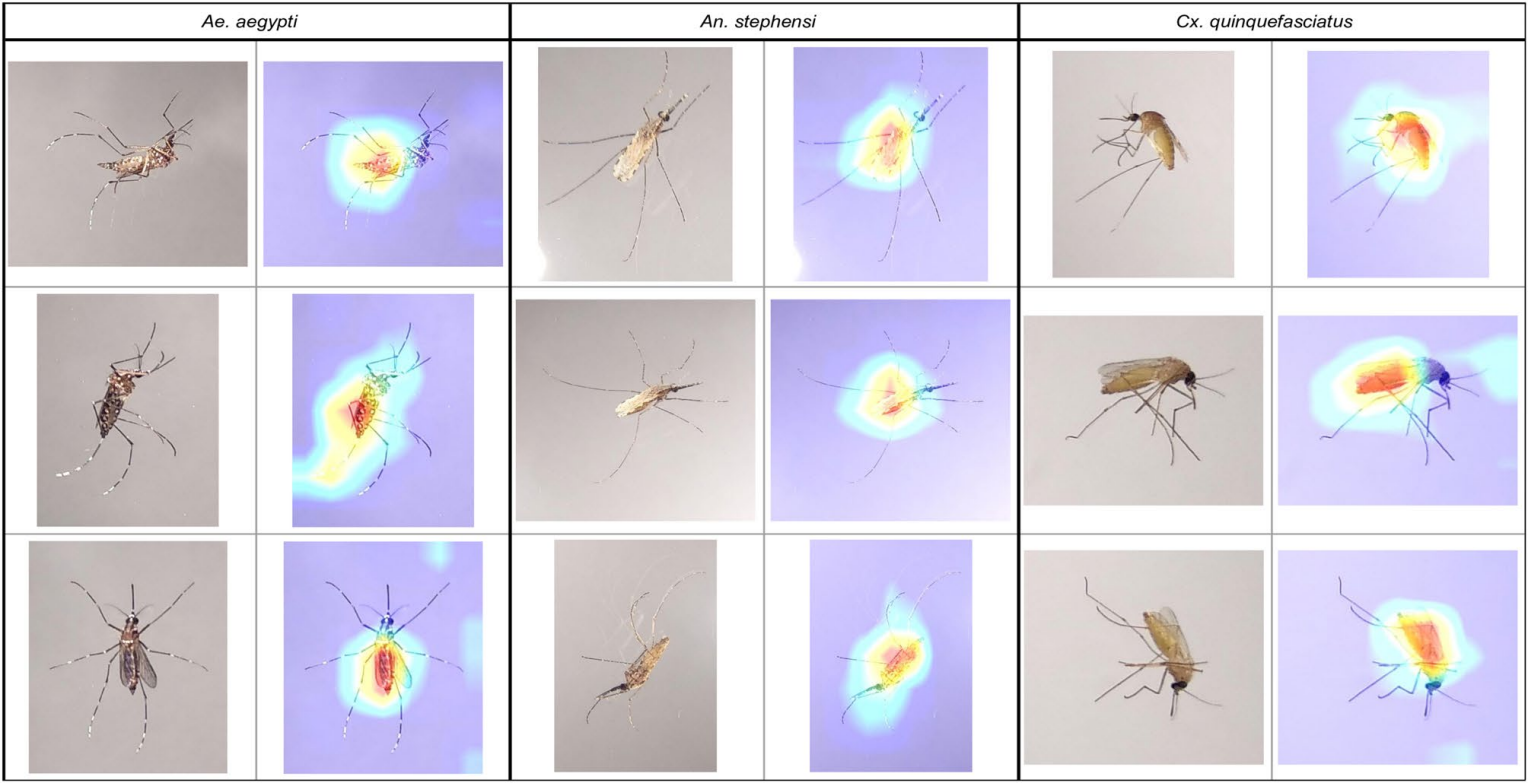
Gravid gonotrophic stage images with Grad-CAM per species for the *EfficientNet-B0* model.

Figures 3, 4, 5, and 6 show specific instances of Grad-CAM outputs on test images based on classifications made by our *EfficientNet-B0* model, organized by gonotrophic stage and species. Results demonstrated that the redder pixels are indeed concentrated in and around the abdomen in all instances. This provides a high degree of confidence that the *EfficientNet-B0* model has learned correctly from the right anatomical components in an image, and we can hence explain the classification of gonotrophic stages in a mosquito. We mention here that while only some instances are presented in Figs. 3, 4, 5 and 6, the results are indeed generalizable.

## Discussions

The surveillance and control of mosquito vectors is a critical aspect of epidemiology, but the process is fraught with obstacles. The standard surveillance practice is to lay mosquito traps in an area of interest, after which the trapped mosquitoes—sometimes hundreds per trap—are brought to a lab and spread out on a board that is light-colored for visual inspection one-by-one (to identify to species, gonotrophic stage, etc.). Sometimes, a microscope is needed too. This process is arduous, manual, and time-consuming. Additionally, across the globe, entomology is a profession for which expertise is increasingly difficult to find and sustain. There is a clear need to automate the surveillance process, with practical ramifications elaborated below^13,14^.

Knowing the abundance of mosquitoes in each gonotrophic stage is important for a variety of assessments, including near-time forecasting of the population of vector mosquitoes (via knowing the abundance of gravid mosquitoes), the effectiveness of eradication strategies in controlling vectors, conduciveness of local climactic factors for reproduction (gleaned via the ratio of the number of gravid to fully fed mosquitoes), and propensity for diseases to spread in any area during any outbreak (based on the number of blood-fed mosquitoes).

For the specific case of malaria (and also for other mosquito-borne diseases), it has been shown that understanding the gonotrophic stages of mosquitoes has vital importance for disease control and associated environmental impact^3,4^. Specifically, it has been shown that being aware of the timing of blood meals and egg laying will enable highly targeted eradication strategies to reduce mosquito populations and hence diseases. This is because a targeted plan to use pesticides across space and time will not only suppress mosquito populations and the spread of disease effectively, but it will also lower costs and the associated environmental impact.

Mosquito fecundity is primarily determined by their neurosecretory system, the amount of blood they consume, and local climatic circumstances^15,16^. If any of these conditions are unfavorable, fertility decreases. Hence, the effect of a single factor on fecundity can be determined, after controlling for other variables, by determining the relative abundance of mosquitoes in various gonotrophic stages. In addition, given species-specific and gonotrophic stage knowledge, public health experts can compare the fecundity of different mosquito species to gain a deeper understanding of the differences in their reproductive biology.

Knowledge of the gonotrophic stages is also critical to other facets of mosquito-borne disease epidemiology. For example, a fully fed mosquitoes are required for enzyme-linked immunosorbent assays (ELISA), to identify human blood meals in mosquitoes^17^, and semi-gravid mosquitoes are required for cytogenetic analysis to assess chromosomal mutations^18^. Furthermore, since a mosquito needs to have consumed a blood meal to carry pathogens, an automated and rapid mechanism to classify a fed mosquito from an unfed one will enhance operational efficiency in determining the presence or absence of pathogens in any specific mosquito during outbreaks.

Beyond merely helping entomologists save time in gonotrophic stage identification, the impact of our paper extends onto two novel avenues. The first is leveraging image data generated by citizen science (aka. community science). Our team is now close partners with three well-established platforms that the general public uses to upload mosquito observations. These platforms are Mosquito Alert^19^, iNaturalist^20^, and GLOBE Observer’s Mosquito Habitat Mapper^21^. Via these partnerships, we work with volunteers across Africa, the Americas, and Europe to train citizen scientists on best practices for recording and uploading mosquito observations from smartphones. Furthermore, utilizing Open Geospatial Consortium standards, we have harmonized data streams from all of these platforms to facilitate interoperability and utility for experts and the general public. This GIS mapping platform, the Global Mosquito Observations Dashboard (GMOD), is accessible at www.mosquitodashboard.org for visualizing and downloading data in multiple tabular and geospatial formats (> 300K observations to date)^22,23^. We are currently integrating computer vision algorithms that we have designed and validated in prior work^22–25^ to process images from these citizen science platforms for species identification (and soon for gonotrophic stage identification). Notably, most mosquito images uploaded by citizen scientists are taken indoors with a light-colored wall background when the mosquito is resting. This is also a reason why the images of mosquitoes in our dataset were taken on a gray- or white-colored background.

The second novel practical impact of our work lies in augmenting AI technologies that we and multiple other groups are designing to identify mosquito species automatically, thereby eliminating the need for expert human involvement^26–28^. While in some technologies, a mosquito must be emplaced in an imaging system^26^, in other technologies, mosquito images are captured in flight inside the trapping chamber^29^. In either case though, the background is light-colored to provide appropriate contrast. Ultimately, the algorithms shared in our paper (Data availability) can enable novel tools that harness the power of both AI and the general public, as they upload images from which we can now not only identify vector mosquitoes but also their gonotrophic stages, with greater utility for mosquito surveillance and control.

## Conclusions

In this study, we develop computer vision approaches to automate the determination of gonotrophic stages from mosquito images. Our data came from mosquitoes distributed across three important species: *Ae. aegypti*, *An. stephensi*, and *Cx. quinquefasciatus*. A total of 139 mosquitoes were raised in two separate facilities, and they went through the four gonotrophic stages: unfed, fully fed, semi-gravid, and gravid. Using multiple smartphones, we then captured 1959 photographs of these mosquitoes against a plain background. Following that, we trained and tested four diverse but popularly used AI model architectures and implemented explainable AI techniques (t-SNE, Grad-CAMs) to validate their outcomes. Overall, the *EfficientNet-B0* model gave the best performance when combining model accuracy, model size, distinguishable t-SNE plots, and correct Grad-CAMs.

To the best of our knowledge, our contributions in this paper are the first towards automating the process of determining the gonotrophic stage of a mosquito using computer vision techniques. We believe that our method provides novel tools for entomologists, citizen-science platforms, and image-based mosquito surveillance. With the increasing spread and resurgence of mosquito-borne diseases across the globe (e.g., the first local transmission of malaria in the US in two decades this summer), our study assumes critical and urgent significance.

## Methods

### Generation of image database and augmentation

The images comprising our dataset came from mosquitoes raised in captivity in two separate labs. One lab is in South India, and the other is in the US. The mosquitoes raised in South India belonged to three species: *Ae. aegypti, An. stephensi*, and *Cx. quinquefasciatus*. Mosquitoes were fed with chicken and sheep blood in India and the US respectively. It took about two minutes for the mosquitoes to reach a fully fed state. After this, the mosquitoes took about 24 hours to move from one stage in the gonotrophic cycle to the next. At each stage, the mosquitoes were visually observed by entomological experts to determine the correct stage. Please note that after visual identification, live mosquitoes were emplaced in test tubes and anesthetized using a few drops of diethyl ether added to the cotton plug of the test tubes. Within a minute, mosquitoes were anesthetized. The mosquitoes were then photographed over a plain grey or white background with multiple smartphones. The background was chosen specifically since (a) entomologists today emplace mosquitoes on a light-colored platform for identification; (b) citizen-uploaded images of mosquitoes in portals today are predominantly taken indoors on a light colored background; and (c) the light-colored background provides the highest contrast. The reason for taking images via multiple smartphones was to introduce noise that commonly occurs in real life due to diversity across cameras, and is a standard procedure in computer vision. This same image-capturing procedure was also followed for the mosquitoes raised in the US, except that these were only *An. stephensi* mosquitoes, and the photographs taken were for the unfed and semi-gravid stages only. The final dataset contained 579 images of unfed female mosquitoes, 521 images of fully fed mosquitoes, 438 images of semi-gravid mosquitoes, and 421 images of gravid mosquitoes across the three species (see Table 1). It is important to note here that a mosquito that was photographed in one stage was not used for photographs taken in another stage. In other words, photographs of a single mosquito specimen were taken for only one gonotrophic stage in our dataset. This alleviates pseudo-replication concerns in our dataset.

**Figure 7:**
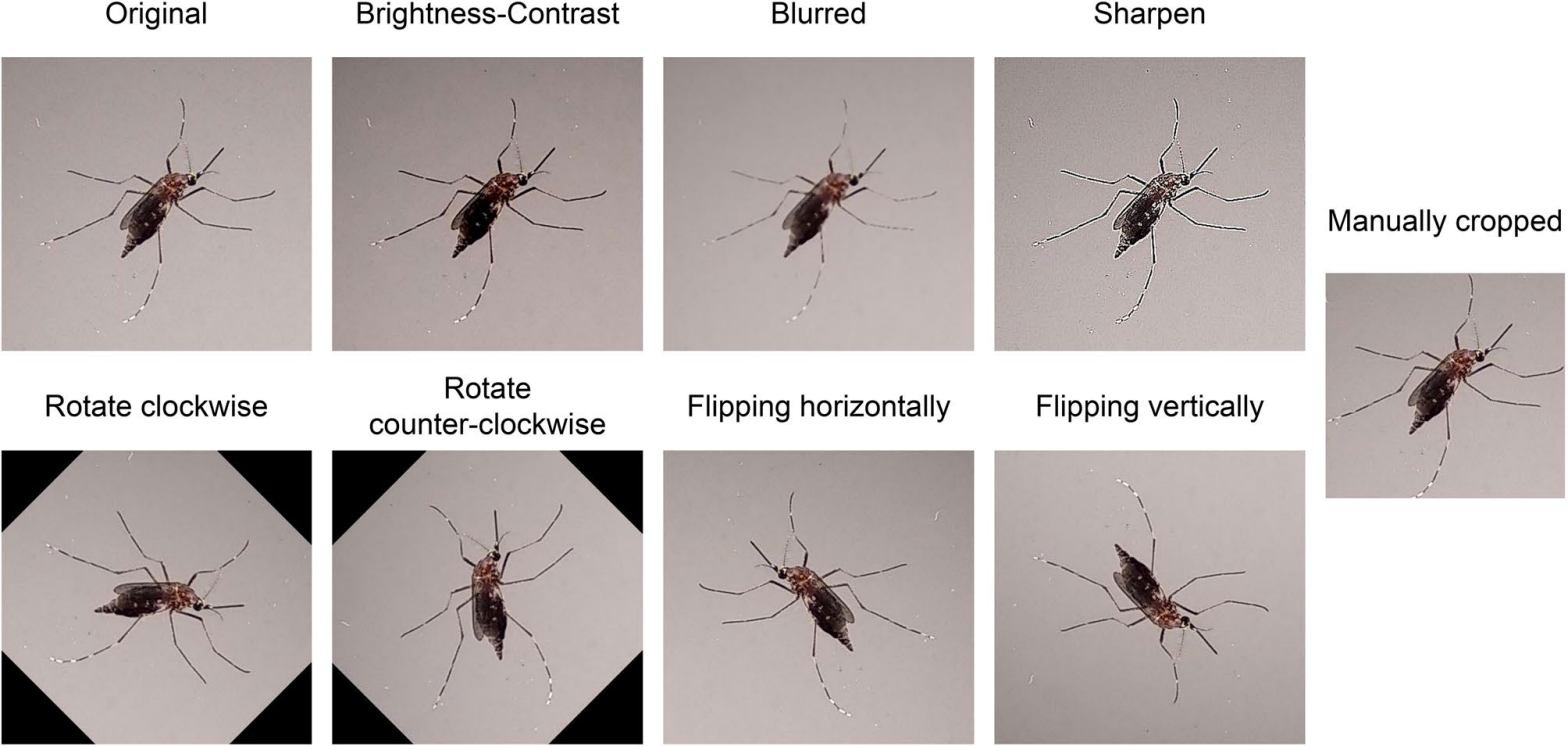
Augmented images for a sample mosquito image.

Once the images were generated, the entire image and species dataset was split into training, validation, and testing sets in the proportion of $80\%$ (1504 images), $10\%$ (221 images), and $10\%$ (234 images), respectively. Images in the training set were augmented, which is a standard step before developing AI models. The idea here is to introduce sufficient diversity to the training samples, so that the model learns to better ignore noninformative variation during practical use, and is not over-fitted. To augment the training images (i.e., add diversity to the 1504 training images), we used eight methods that are standard in image processing—rotating clockwise (a) and counter-clockwise (b), flipping horizontally (c) and vertically (d), changing blurriness (e) and sharpness (f), altering brightness randomly from 5 to 20$\%$ (g), and manually cropping images to extract only the mosquito body (h). Figure 7 shows eight augmented images with the original image of a sample mosquito. After augmentation, the number of training samples per class was increased by a factor of eight. The images in the validation and testing sets were not augmented.

### Our deep neural network architectures to classify gonotrophic stages of mosquitoes

#### Deep neural networks architectures rationale

In this paper, we trained and validated four distinct deep neural network architectures for gonotrophic stage classification—*ResNet50*, *MobileNetV2*, *EfficientNet-B0*, and *ConvNeXtTiny*. All these architectures are popular in the literature and are sufficiently diverse. The *ResNet50*^5^ architecture employs a series of residual blocks, each containing convolutional layers and utilizing a bottleneck design to optimize computation. *ResNet50*^5^ addresses the vanishing gradient problem by introducing shortcut connections (skip connections) which help in training very deep neural networks. However, this model can be computationally very intensive and memory-consuming due to the increased depth and the necessity to store intermediate activations for the skip connections. This can make it challenging to deploy on resource-constrained devices or platforms. A lighter-sized model well-suited for execution on embedded devices like smartphones is *MobileNetV2*^6^, which utilizes depth-wise separable convolutions, significantly reducing the number of parameters and computations compared to traditional convolutional layers. This makes it highly efficient for mobile and embedded devices, allowing for faster inference and lower memory requirements. The efficiency gain achieved in *MobileNetV2* comes at the cost of some loss in accuracy compared to larger and more computationally intensive models. As a trade-off between accuracy and computation cost, we chose the *EfficientNet-B0*^7^ model for training and validation. It has achieved state-of-the-art performance across various tasks while requiring fewer parameters compared to other architectures. Instead of independently scaling the width, depth, and resolution of the network, *EfficientNet-B0* scales all three aspects simultaneously and uniformly using scaling coefficients. Thus it strikes a superior balance between model size, computational efficiency, and accuracy, making it highly efficient for practical applications and deployment. Apart from these three convolutional neural networks, we finally trained and validated *ConvNeXtTiny*^8^, a recent neural network inspired by the concepts of Vision Transformers^9^ (which are very state-of-the-art now, but heavy). It employs a technique known as depth-wise convolution and it is a distinct approach to image processing where the network analyzes various segments of the image independently. This method effectively cuts down on the required computational workload while preserving accuracy.

#### Optimization of hyperparameters

As is customary in developing deep neural network architecture, hyperparameters are determined by multiple rounds of training and validation on the dataset^30^. Critical hyperparameters tuned in our neural network architecture are presented in Table 6. Please note that Table 6 lists those hyperparameters that we used for training and validating the *EfficientNet-B0* model although they were very similar for the other three architectures too.

**Figure 8:**
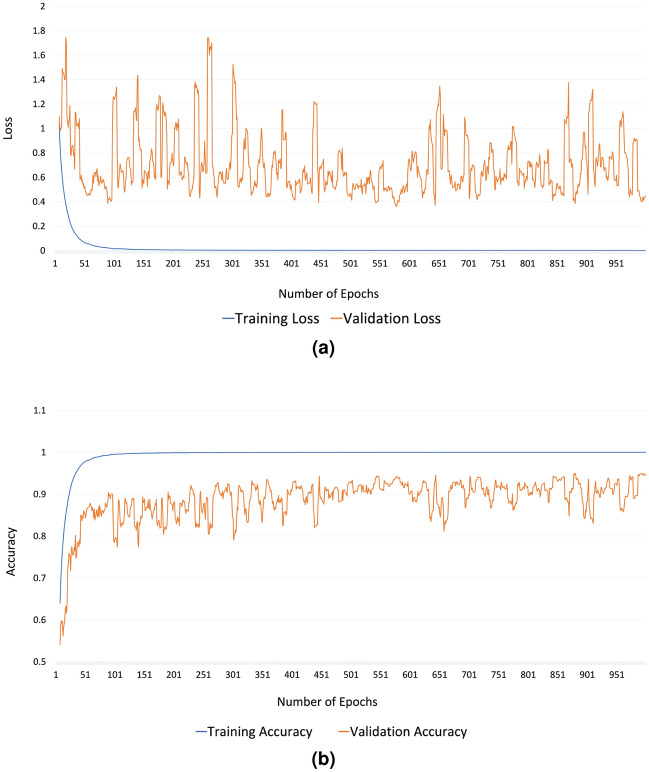
Plotting the loss (**a**) and accuracy (**b**) for each epoch in training and validation of *EfficientNet-B0*.

- **Resized images:** To maintain image consistency, we must resize them. As we collected data from numerous cell phones for our problem, we downsized each input image to $224 \times 224 \times 3$ pixels, regardless of its actual dimension for consistency. This enables us to achieve faster training without loss of image quality. We standardized the RGB value of each pixel in the image by dividing it by 255.
- **Optimizer:** In this work, the Adam (Adaptive Moment Estimation)^31^ optimization algorithm was utilized. This technique enables adaptive learning rates for weights across architectural layers, so that lower rates are allocated to weights receiving larger updates and higher rates are given to weights receiving smaller updates. The exponential decay rates for the first and second moment estimations ($\beta _1$ and $\beta _2$) are set to 0.89 and 0.999 respectively.
- **Loss functions:** In this study, we utilized the categorical cross-entropy loss function. This function minimizes the difference between the expected probability function and the actual probability function. This is in contrast to other loss functions, such as focal loss and triplet loss, which perform better when variations in terms of the complexity of entities inside classes and their inter-variabilities are greater, neither of which is true for our situation.
- **Fine-tuning of the architecture and compensating for overfitting:** For fine-tuning, we initially froze the layers of the base model (all the layers except for the the *dense* layers we appended to the bottom of the architecture (see Table 5)) with the weights of a pre-trained model that was trained on the ImageNet^32^ of 14 million images of 20,000 categories. Since the model was already trained on a large dataset, these weights were already highly optimized. That is why we only trained the last 14 layers of the model with a higher learning rate ($1e-3$). After training with 500 epochs, we unfroze all layers and again trained the model with a smaller learning rate ($1e-5$) so that the change in weights would be smaller. Within 500 epochs (in total 1000), the model reached the best optimization. Figure 8 shows the loss and accuracy for each iteration during training and validation of the *EfficientNet-B0* model. We have used Python DL libraries (*keras*^33^, *tensorflow*^34^) to implement the codes and trained on an Intel Xeon E5-2620 v4 processor with 128GB GPU memory.

**Table 5:** EfficientNet-B0 model architecture with layer information (input and output sizes).

Layer	Size in	Size out
*Top_activation* (layer 236 in *EfficientNet-B0*)	(None, 7, 7, 1280)	(None, 7, 7, 1280)
*Average_pooling2d*	(None, 7, 7, 1280)	(None, 1, 1, 1280)
*Dense_1*	(None, 1, 1, 1280)	256
*Dense_2*	256	128
*Dense_3*	128	128
*Concatenate*	(Dense_1, Dense_2, Dense_3)	512
*Softmax*	512	4

**Table 6:** Values of critical hyperparameters in training the *EfficientNet-B0* model.

Hyperparameter	Value
Number of layers	250
Learning rate	1e−3 for 1–500 epochs
	1e−5 for 501–1000 epochs
Optimizer	Adam
Batch size	16
Loss function	Categorical cross-entropy
Number of epochs	1000

## Related work

Mosquito surveillance and control are critical tasks in epidemiology. There is always a need to enhance the speed and scale of these activities, especially with rising cases of mosquito-borne diseases across the globe.

In the past decade or so, citizen-science platforms such as iNaturalist^20^, Mosquito Alert^19^, and Mosquito Habitat Mapper^21^ have been deployed with great success^22,35^, enabling non-experts to take and upload photographs of mosquitoes that they encounter in nature. Experts can then identify and analyze these data, hence providing a new source of surveillance information beyond the limits of traditional trapping methods. In addition, rapid advancements in AI techniques have also enabled numerous image processing methods for mosquito identification. Specific problems addressed by such studies are presented below in limited detail.

Goodwin et al.^26^ presented a method for identifying mosquito species using convolutional neural networks (CNNs) and a multitiered ensemble model. The approach utilized deep learning techniques to analyze mosquito images and accurately classify 67 mosquito species. Kittichai et al.,^36^ focused on utilizing the well-known deep learning techniques: you-only-look-once (YOLO) algorithm^37^ for the identification of both mosquito species and gender. The YOLO algorithm, with its ability to handle complex and challenging visual data, aided in accurately identifying and classifying mosquito vectors. Kittichai proposed to concatenate two YOLO v3^38^ models and was able to show optimal performance in mosquito species and gender classification. Finally, our prior work has demonstrated the utility of CAMs in the identification of mosquito species^22,24^.

To the best of our knowledge, there is no work yet in the literature on automating the determination of gonotrophic stages in a mosquito, hence making the contributions in this paper unique and practically impactful.

## Data Availability

The source codes and dataset for this work can be found in the GitHub repository: https://github.com/FarhatBuet14/Classifying-stages-in-the-gonotrophic-cycle-of-mosquitoes.

## References

[1] Yee DA (2022). Robust network stability of mosquitoes and human pathogens of medical importance. Parasites & Vectors.

[2] Malaria, unicef: Status update on children. 25 april 2020. https://data.unicef.org/resources/malaria-snapshots-sub-saharan-africa-and-impact-of-covid19 (2020).

[3] Ferguson HM (2010). Ecology: A prerequisite for malaria elimination and eradication. PLoS Med..

[4] Williams, J. & Pinto, J. Training manual on malaria entomology for entomology and vector control technicians (basic level). *USAID. Washington, DC* **78** (2012).

[5] He, K., Zhang, X., Ren, S. & Sun, J. Deep residual learning for image recognition. In *Proceedings of the IEEE Conference on Computer Vision and Pattern Recognition*, 770–778 (2016).

[6] Sandler, M., Howard, A., Zhu, M., Zhmoginov, A. & Chen, L.-C. Mobilenetv2: Inverted residuals and linear bottlenecks. In *Proceedings of the IEEE Conference on Computer Vision and Pattern Recognition*, 4510–4520 (2018).

[7] Tan, M. & Le, Q. Efficientnet: Rethinking model scaling for convolutional neural networks. In *International Conference on Machine Learning*, 6105–6114 (PMLR, 2019).

[8] Liu, Z. *et al.* A convnet for the 2020s. In *Proceedings of the IEEE/CVF Conference on Computer Vision and Pattern Recognition*, 11976–11986 (2022).

[9] Dosovitskiy, A. *et al.* An image is worth 16x16 words: Transformers for image recognition at scale. arXiv preprint arXiv:2010.11929 (2020).

[10] Van der Maaten, L. & Hinton, G. Visualizing data using t-sne. *J. Mach. Learn. Res.* **9** (2008).

[11] Selvaraju, R. R. *et al.* Grad-cam: Visual explanations from deep networks via gradient-based localization. In *Proceedings of the IEEE International Conference on Computer Vision*, 618–626 (2017).

[12] Kullback S, Leibler RA (1951). On information and sufficiency. Ann. Math. Stat..

[13] Yamany AS (2012). Studies on the development of the ovaries of the malaria mosquitoes (anopheles pharoensis). J. Vaccines Vaccin..

[14] Martínez-de la Puente J, Ruiz S, Soriguer R, Figuerola J (2013). Effect of blood meal digestion and DNA extraction protocol on the success of blood meal source determination in the malaria vector anopheles atroparvus. Malar. J..

[15] Detinova, T. S., Bertram, D. S., Organization, W. H. *et al.**Age-Grouping Methods in Diptera of Medical Importance, with Special Reference to Some Vectors of Malaria* (World Health Organization, 1962). 13885800

[16] Christophers S (1911). The development of the egg follicles in anophelines. Paludism.

[17] Edrissian GH, Manouchehry A, Hafizi A (1985). Application of an enzyme-linked immunosorbent assay (elisa) for determination of the human blood index in anopheline mosquitoes collected in iran. J. Am. Mosq. Control Assoc..

[18] Bellini, R. *et al.* Use of the sterile insect technique against aedes albopictus in Italy: First results of a pilot trial. In *Area-Wide Control of Insect Pests: From Research to Field Implementation*, 505–515 (Springer, 2007).

[19] Citizen science to investigate and control disease-carrying mosquitoes. http://www.mosquitoalert.com/en/. Accessed: 2023–05–22.

[20] A community for naturalists $$\cdot$$ inaturalist. https://www.inaturalist.org/. Accessed: 2023–05–22.

[21] The globe observer mosquito habitat mapper. https://observer.globe.gov/toolkit/mosquito-habitat-mapper-toolkit. Accessed: 2023–05–22.

[22] Carney RM (2022). Integrating global citizen science platforms to enable next-generation surveillance of invasive and vector mosquitoes. Insects.

[23] Uelmen Jr, J. A., *et al.* Global mosquito observations dashboard (GMOD): Creating a user-friendly web interface fueled by citizen science to monitor invasive and vector mosquitoes. *Int. J. Health Geogr.* **22**, 28 (2023). 10.1186/s12942-023-00350-7 PMC10612222 37898732

[24] Minakshi, M. *et al.* Automating the surveillance of mosquito vectors from trapped specimens using computer vision techniques. In *Proceedings of the 3rd ACM SIGCAS Conference on Computing and Sustainable Societies*, 105–115 (2020).

[25] Minakshi M, Bharti P, Bhuiyan T, Kariev S, Chellappan S (2020). A framework based on deep neural networks to extract anatomy of mosquitoes from images. Sci. Rep..

[26] Goodwin A (2021). Mosquito species identification using convolutional neural networks with a multitiered ensemble model for novel species detection. Sci. Rep..

[27] Goodwin A (2020). Development of a low-cost imaging system for remote mosquito surveillance. Biomed. Opt. Express.

[28] Chellappan, S., Minakshi, M., Bharti, P. & Carney, R. M. Systems and methods for classifying mosquitoes based on extracted masks of anatomical components from images (2023). US Patent App. 17/462,809.

[29] Chellappan, S., Saddow, S. E., Carney, R. M., Wolfram, B. & Weston, M. Smart mosquito trap for mosquito classification (2022). US Patent App. 17/496,563.

[30] Brownlee, J. How to grid search hyperparameters for deep learning models in python with keras. *línea]. Disponible en: https://machinelearningmastery. com/grid-search-hyperparameters-deep-learning-models-python-keras* (2016).

[31] Zhang, Z. Improved adam optimizer for deep neural networks. In *2018 IEEE/ACM 26th International Symposium on Quality of Service (IWQoS)*, 1–2, 10.1109/IWQoS.2018.8624183 (2018).

[32] Deng, J. *et al.* Imagenet: A large-scale hierarchical image database. In *2009 IEEE Conference on Computer Vision and Pattern Recognition*, 248–255 (IEEE, 2009).

[33] Chollet, F. *et al.* Keras. https://keras.io (2015).

[34] The TensorFlow Authors. Tensorflow: Large-scale machine learning on heterogeneous systems. https://www.tensorflow.org/ (2015).

[35] Sousa, L. B. *et al.* Methodological diversity in citizen science mosquito surveillance: a scoping review. *Citizen Science: Theory and Practice* **7** (2022).

[36] Kittichai V (2021). Deep learning approaches for challenging species and gender identification of mosquito vectors. Sci. Rep..

[37] Redmon, J., Divvala, S., Girshick, R. & Farhadi, A. You only look once: Unified, real-time object detection. In *Proceedings of the IEEE Conference on Computer Vision and Pattern Recognition*, 779–788 (2016).

[38] Redmon, J. & Farhadi, A. Yolov3: An incremental improvement. arXiv preprint arXiv:1804.02767 (2018).

